# Autophagy flux in critical illness, a translational approach

**DOI:** 10.1038/s41598-019-45500-9

**Published:** 2019-07-24

**Authors:** Nicolas Tardif, Franck Polia, Inga Tjäder, Thomas Gustafsson, Olav Rooyackers

**Affiliations:** 1 Division of Perioperative Medicine and Intensive Care, Karolinska University Hospital, Huddinge, Sweden; 2 Anesthesiology and Intensive Care, Department of Clinical Science Intervention and Technology (CLINTEC), Karolinska Institutet, Huddinge, Sweden; 3 Division of Clinical Physiology, Department of Laboratory Medicine, Karolinska Institutet, and Unit of Clinical Physiology, Karolinska University Hospital, Stockholm, Sweden

**Keywords:** Macroautophagy, Translational research

## Abstract

Recent clinical trials suggest that early nutritional support might block the induction of autophagy in critically ill patients leading to the development of organ failure. However, the regulation of autophagy, especially by nutrients, in critical illness is largely unclear. The autophagy flux (AF) in relation to critical illness and nutrition was investigated by using an *in vitro* model of human primary myotubes incubated with serum from critically ill patients (ICU). AF was calculated as the difference of p62 expression in the presence and absence of chloroquine (50 µM, 6 h), in primary myotubes incubated for 24 h with serum from healthy volunteers (n = 10) and ICU patients (n = 93). We observed 3 different phenotypes in AF, non-altered (ICU non-responder group), increased (ICU inducer group) or blocked (ICU blocker group). This block was not associate with a change in amino acids serum levels and was located at the accumulation of autophagosomes. The increase in the AF was associated with lower serum levels of non-essential amino acids. Thus, early nutrition during critical illness might not block autophagy but could attenuate the beneficial effect of starvation on reactivation of the autophagy process. This could be of clinical importance in the individual patients in whom this process is inhibited by the critical illness insult.

## Introduction

Critically ill patients treated in an Intensive Care Unit (ICU) often have organ failure already at their admittance or will develop it early during their stay in the unit. When vital organs fail, the patients will not survive without organ support and despite treatment the mortality rates are high and increase with the number of organs failing. Although not a vital organ, the loss of skeletal muscle mass and function is one of the most characteristic features during critical illness. The loss of muscle mass is related to the patient’s mortality, morbidity, response to treatment and speed of recovery^1^. Since nutrition is known to preserve muscle mass during health, aggressive feeding strategies with the use of both enteral and parenteral routes of nutrition have been commonly used in ICUs. Earlier observational studies demonstrate underfeeding to be associated with less favorable outcomes, i.e. longer duration of mechanical ventilation, ICU stay and recovery time^2,3^. On the other hand, the two largest randomized clinical trials performed in the ICU in 2014 (EPaNIC trial) and in the pediatric ICU in 2016 (PEPaNIC trial) complicated the nutritional consensus since these studies indicated that early supplemental parenteral nutrition may have a negative impact on organ failure and thereby prolong ICU stay^4,5^. Post hoc analysis of these two trials suggested that the delayed recovery from organ failure associated with the early administration of parenteral nutrition was explained by a suppression of autophagy. However direct evidence of the role of autophagy in organ failure development and the impact of nutrition in this mechanism is lacking^4,6,7^.

A growing body of evidence supports autophagy to be essential in the recovery from critical illness-induced organ failure. In an experimental rodent model using cecal ligation and puncture (CLP), an initial increase in autophagy was observed followed by a later block in the autophagy flux in the liver of the septic animals^8,9^. Importantly, inhibition of autophagy increased mortality rates^9^ and when reactivated either by rapamycin treatment or by a transgenic overexpression of Microtubule associated protein 1 light chain 3 (LC3) survival rates increased^10,11^. Moreover, in septic patients a higher risk of death is associated to the genetic polymorphism of the human immunity-related GTPase M, a protein regulating the autophagy process known to participate in the defense against invading pathogens^12^.

Autophagy is a self-degradative process of importance for balancing sources of energy at critical times such as starvation and infections. Autophagy is a dynamic, multi-step process, which could be resumed in 3 mains steps. The first step is the creation of a double membrane, called phagophore, around the substrate that is to be degraded (initiation step). In the second step, the phagophore is closed, and an autophagosome is formed (elongation step). In the last step, the autophagosome fuses with a lysosome leading to an autolysosome (fusion step). Thereafter the cellular substances carried by the autophagic vacuoles are degraded by the lysosomal enzymes. Unfortunately, in clinical studies autophagy can only be assessed by measuring the static expression of autophagy markers, since measurements of the flux of autophagy i.e. *in vivo* measurements, are technically not possible in humans. In ICU patients a higher expression of nucleoporin p62 (p62), a marker of autophagy blockage^13^ has been observed in liver and skeletal muscle^7,14^. Also the autophagy block suggested in the ICU patients receiving early parenteral nutrition, was associated with a lower LC3II to LC3I ratio, a surrogate marker of autophagosome formation^7^. Thus, autophagy is demonstrated to be essential in the recovery from critical illness-induced organ failure but plausible reasons and mechanisms of dysregulation in critically ill patients remain largely unknown as well as the potential deleterious effect of early nutrition, especially the dietary amino acids.

To be able to measure the autophagy flux in relation to critical illness and nutrition, an approach using an *in vitro* model of human primary myotubes incubated with serum from ICU patients was used in the present study. Our results show that autophagy was blocked in a subpopulation of ICU patients, but this was not dependent on the serum level of amino acids. Lower levels of amino acids are however associated with a higher autophagy flux. Hence the results from the PEPaNIC and EPaNIC trial might be reinterpreted as not being a deleterious effect of early nutrition via a block of autophagy but to an attenuation of the beneficial effect of starvation on reactivation of the autophagy process blocked by the illness.

## Results

### Serum from ICU patients induce different autophagy flux responses

**Figure 1:**
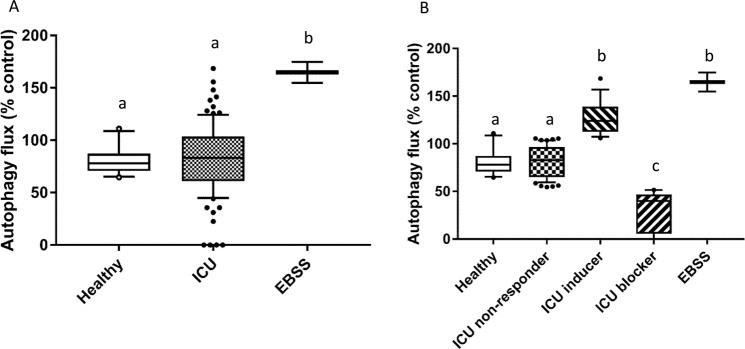
Serum from ICU patients induce different phenotype of autophagy flux. The flux of autophagy was calculated from the difference in p62 expression measured in the presence and absence of chloroquine (6 h, 50 μM) in human primary myotubes. The protein expression of p62 was measured *in situ* by in cell western. (**A**) Autophagy flux measured in human primary myotubes incubated for 24 h with 10% of serum from ICU patients (n = 93) and healthy volunteers (n = 10). (**B**) The patient cohort was dived into three groups based on the degree of the serum-induced change in autophagy flux e.g. ICU non-responder (n = 59), ICU inducer (n = 18) and ICU blocker (n = 16). The threshold value used to split the cohort was defined from the measured autophagy flux induced by serum from the healthy volunteers ± 2 SD. Results are presented as medians, with box plots representing 25th and 75th percentiles as boxes and 10th and 90th percentiles as whiskers. Human primary myotubes were starved for 6 h in Earle’s Balanced Salt Solution (EBSS) as a positive control for an increase in autophagy flux. The result presented are from 5 independent experiments. Statistical difference was assessed by the non-parametric Kruskal-Wallis test with a ≠ b ≠ c, p < 0.05. Healthy: healthy volunteers (n = 10); ICU: All ICU patients (n = 93); ICU non-responder (n = 59): group of patients from whom the serum do not induce a change in autophagy flux when compared to the healthy group; ICU inducer (n = 18): group of patients from whom the serum induce an increase in autophagy flux; ICU blocker (n = 16): group of patients from whom the serum induce a block in the autophagy flux.

Previous *in vitro* experimental models using cells incubated with serum from ICU patients have reproduced some of the phenotypes observed in these patients, i.e. muscle atrophy with loss of myosin and mitochondrial dysfunction^15,16^. More recently, an activation of autophagy in myotubes induced by serum from cachectic cancer patients has been shown^17^. In our study, the effect of serum from ICU patients (n = 93) on the autophagy flux was assessed *in vitro* in human primary myotubes. The autophagy flux was calculated by measuring the *in situ* expression of p62 in myotubes exposed to 10% serum of patients and healthy volunteers in the presence or absence of chloroquine (6 h, 50 µM). On average, serum from the ICU patients did not induce a change in the autophagy flux when compared with serum of the healthy volunteers (82 ± 33 vs. 81 ± 13 A.U, Fig. 1A). However, we observed a very large variation in the autophagy flux induced by the patients’ serum (CV = 41%) compared to the variation observed in the healthy volunteers group (CV = 16%). A large dispersion is a well-known fact when measuring different variables in a cohort of ICU patients. This could be explained by various reasons, such as various disease aetiology or higher severity of disease but could also relate to an individual difference in the autophagy response to disease-induced stress or to specific treatments. If the latter, this could contain important information leading to better understanding of the autophagy process in ICU patients and thereby provide clinical tools to facilitate this process in the disease recovery phase. Thus, instead of focusing on the average response of the whole group, the patient cohort was dived into three groups based on the degree of the serum-induced change in the autophagy flux e.g. ICU non-responder (n = 59), ICU inducer (n = 18) and ICU blocker (n = 16). The threshold value used to split the cohorts was defined from the autophagy flux measured in myotubes incubated with serum from the healthy volunteers ± 2 SD (Fig. 1B). The autophagy flux observed in the ICU inducer group (127 ± 18 A.U) was not significantly different from the starvation condition (165 ± 10 A.U, Fig. 1B).

### The block of autophagy is associated with a higher autophagosome accumulation

**Figure 2:**
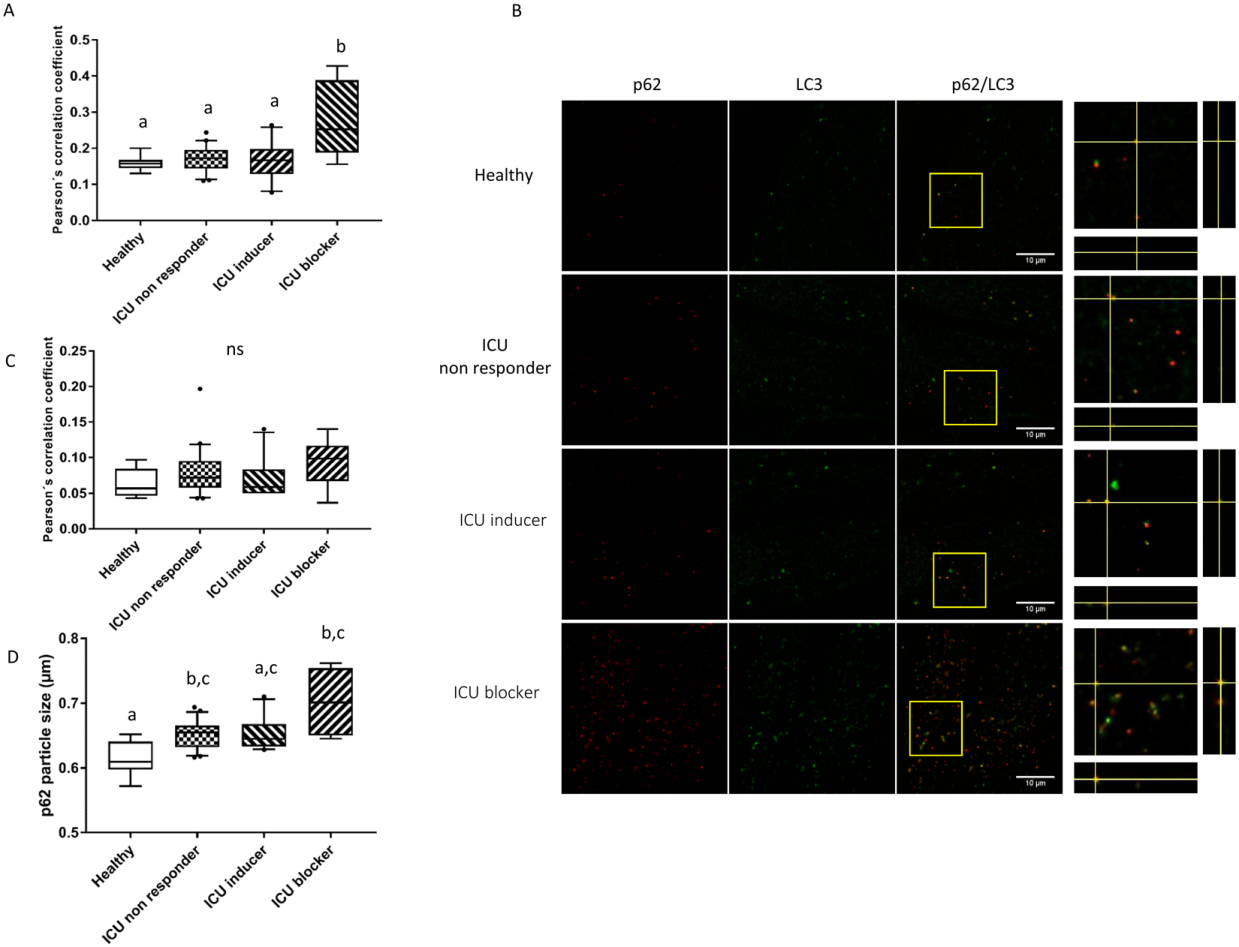
Autophagy flux block is associated with a higher colocalization of LC3 with p62. Human primary myotubes were incubated with serum from ICU patients for 24 h and then fixed in paraformaldehyde and immuno-stained with p62, LC3B and Mono- and Poly-ubiquitin primary antibodies. Pearson colocalization correlation coefficient of LC3 with p62 (**A**) are presented as medians, with box plots representing 25th and 75th percentiles as boxes and 10th and 90th percentiles as whiskers. Representative images of the p62 and LC3B immunofluorescence signal per groups are presented as merged images and with an orthogonal view representation (**B**). Pearson colocalization correlation coefficient of mono- and poly-ubiquitin with p62 (**C**) and the p62 particles diameter size expressed in microns (**D**) are presented as medians, with box plots representing 25th and 75th percentiles as boxes and 10th and 90th percentiles as whiskers. Statistical difference was assessed by the non-parametric Kruskal-Wallis test with a ≠ b ≠ c, p < 0.05. Healthy: healthy volunteers (n = 10); ICU non-responder (n = 59): group of patients from whom the serum do not induce a change in autophagy flux when compared to healthy group; ICU inducer (n = 18): group of patients from whom the serum induce an increase in autophagy flux; ICU blocker (n = 16): group of patients from whom the serum induce a block in the autophagy.

From a principal point of view, a block in the autophagy flux could be due to either (1) a block in the autophagy induction, leading to an accumulation of protein aggregates and damaged organelles or (2) an impairment of the fusion step, leading to an accumulation of autophagosomes or impaired autolysosomes^13^. Aiming to identify the type of block, colocalization of p62 to LC3 or ubiquitin was measured. p62 is a cargo protein that recruits ubiquitinated protein aggregates via its ubiquitin binding (UBA) domain^18^. LC3 identifies the autophagosome/autolysosome vacuoles and ubiquitined (Ub) protein aggregates. The Pearson colocalization coefficient (PCC) of p62 and LC3 demonstrated a higher degree of colocalization in the ICU blocker group (PCC = 0.28 ± 0.11) compared to the healthy volunteers (PCC = 0.16 ± 0.02, *p* < 0.0001), the ICU inducer group (PCC = 0.17 ± 0.05, *p* < 0.0005; Fig. 2A,B) and the ICU non-responder group (PCC = 0.17 ± 0.04, *p* < 0.0001; Fig. 2A,B**)**. The PCC of p62 and LC3 was also negatively correlate with the autophagy flux (Pearson’s r = − 0.36; *p* = 0.014), indicating that the larger the accumulation, the lower the autophagy flux. No significant change in p62 and Ub colocalization was observed between the groups (Fig. 2C). The size of the vesicles marked with p62 (p62 vesicles) was in the same range as previously described^18^ and since they were all below the µm scale, they are most likely representing autophagosomes/autolysosomes rather than protein aggregates^18–20^. The diameter of the p62 vesicles was larger in the ICU blocker group (0.70 ± 0.05 µm) compared to the healthy volunteers (0.61 ± 0.03 µm, *p* = 0.0005) but not when compared to the ICU non-responder (0.65 ± 0.02 µm, *p* = 0.59) or the ICU inducer groups (0.65 ± 0.02 µm, *p* = 0.48) (Fig. 2D). p62 vesicles were also significantly larger in the ICU non-responder group compared to the healthy volunteer group (*p* = 0.014; Fig. 2D).

### *In vitro* changes in autophagy flux are not associated with clinical outcomes

To identify clinical factors that could be associated with the block in autophagy, the groups’ clinical parameters were compared. Aging has been associated with a lower autophagy flux before^21^, but in the current study this factor could not explain the differences between the groups (Table 1). A trend was observed in the ICU blocker group for a higher severity of disease, assessed by the number of organs failing, SOFA (sequential organ function assessment) and APACHE II (acute physiology and chronic health evaluation) score (Table 1), but these did not reach statistical significance.

**Table 1:** Clinical parameters.

	Healthy (n = 10)	ICU non-responder (n = 59)	ICU inducer (n = 18)	ICU blocker (n = 16)	*p*
Age (years)	49 [44–53]	57 [52–63]	59 [50–69]	56 [46–67]	0.55
Sex (F/M)	(5/5)	(21/38)	(7/11)	(8/8)	0.66
BMI (kg/m^2^)	—	26.8 [25.3–28.2]	27.4 [24.3–30.5]	27.0 [23.3–30.8]	0.95
Length of Stay (days)	—	4.5 [3.2–5.9]	5.2 [2.0–8.3]	5.3 [2.3–8.2]	0.94
SOFA	—	6.3 [5.4–7.2]	5.9 [3.8–8.1]	7.9 [5.6–10.1]	0.26
APACHE	—	17.5 [15.4–19.7]	18.3 [14.6–22.1]	19.5 [16.7–22.3]	0.31
Number of organ failing	—	2.8 [2.5–3.1]	2.9 [2.2–3.7]	3.4 [2.6–4.2]	0.23

Results are expressed as mean and 95% CI in bracket. BMI: Body mass index; SOFA: sequential organ failure assessment score; APACHE: Acute Physiology and Chronic Health Evaluation score.

### Low levels of non-essential amino acids in serum are associated with a high autophagy flux

**Figure 3:**
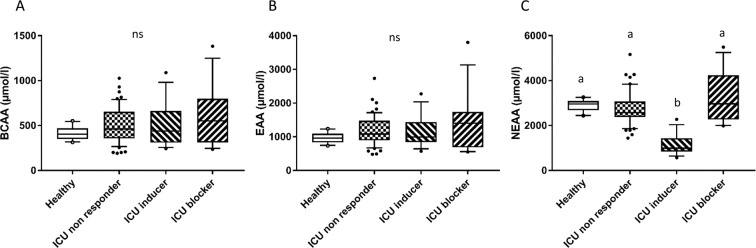
Serum concentrations of amino acids in patients and healthy volunteers. Amino acids were measured in serum of the ICU patients (n = 93) and the healthy volunteers (n = 10) by high performance liquid chromatography. (**A**) The sums of the branched chain amino acids (BCAA; leucine, isoleucine and valine), (**B**) the essential amino acids (EAA; BCAA, phenylalanine, threonine, tryptophan, methionine, lysine and histidine) and (**C**) the non-essential amino acids are expressed as µmol/L and presented as medians, with box plots representing 25th and 75th percentiles as boxes and 10th and 90th percentiles as whiskers. Statistical difference was assessed by the non-parametric Kruskal-Wallis test with a ≠ b, p < 0.05. Healthy: healthy volunteers (n = 10); ICU non-responder (n = 59): group of patients from whom the serum do not induce a change in autophagy flux when compared to healthy group; ICU inducer (n = 18): group of patients from whom the serum induce an increase in autophagy flux; ICU blocker (n = 16): group of patients from whom the serum induce a block in the autophagy.

As clinical studies in the adult ICU in 2014 (EPaNIC trial) and in the pediatric ICU in 2016 (PEPaNIC trial) indirectly suggested a role of autophagy in organ failure development and importance of nutrients on the autophagy process, the serum levels of nutrients were measured and the type of nutrition defined. Glycaemia was not different between the ICU non-responder group (8.4 ± 2.6 mmol/L), the ICU inducer group (8.7 ± 3.2 mmol/L) and the ICU blocker group (11.6 ± 7.5 mmol/L) (*p* = 0.59). There was no significant difference in the percentage of patients fed with enteral nutrition between the ICU non-responder (19%), ICU inducer (22%) and ICU blocker (22%) groups (p = 0.95). The same was observed for parenteral nutition; 25%, 10% and 11% in the ICU non-responder, ICU inducer and ICU blocker groups respectively (p = 0.28). Serum concentrations of the branch chain amino acids (BCAA) and the essential amino acids (EAA) were not different between the groups (Fig. 3A,B). However, the serum concentrations of the non-essential amino acids (NEAA) were significantly lower in the ICU inducer group (1143 ± 452 µmol/L) compared to the healthy group (2903 ± 273 µmol/L, *p* < 0.0001), the ICU non-responder group (2765 ± 718 µmol/L, *p* < 0.0001) and the ICU blocker group (3210 ± 1152 µmol/L, *p* < 0.0001) (Fig. 3C). Importantly, the sum of all the amino acids present in serum of all individual patients was negatively correlated to the autophagy flux (Pearson’s r = −0.29; *p* = 0.011) Individual amino acids that specifically correlated to the autophagy flux were citrulline (r = −0.35; *p* = 0.0013), glutamate (r = −0.31; *p* = 0.005), glycine (r = −0.30; *p* = 0.006), lysine (r = −0.30; *p* = 0.007) and threonine (r = −0.30; *p* = 0.008).

### Serum from ICU patient induce an accumulation of p62 associated with high aminoacidemia

**Figure 4:**
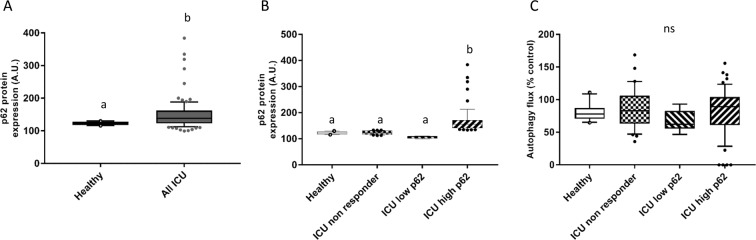
Accumulation of p62 induced by serum of ICU patients is not associated with changes in the autophagy flux. The basal expression of p62 expression induced after 24 h incubation with serum form all ICU patients was measured *in situ* in human myotubes (**A**). The patient cohort was dived into three groups based on the degree of the serum-induced change in basal p62 expression e.g. ICU non-responder (n = 29), ICU low p62 (n = 9) and ICU high p62 (n = 55) (**B**). The threshold value used to split the cohort was defined from the basal p62 expression induced by serum from the healthy volunteers ± 2 SD. Autophagy flux, calculated from the difference in p62 expression measured in the presence and absence of chloroquine (6 h, 50 μM), was not different between p62-based ICU groups (**C**). Results are presented as medians, with box plots representing 25th and 75th percentiles as boxes and 10th and 90th percentiles as whiskers. Statistical difference was assessed by the non-parametric Kruskal-Wallis test with a ≠ b, p < 0.05. Healthy: healthy volunteers (n = 10); All ICU: All ICU patients (n = 93); ICU non-responder (n = 29): group of patients from whom the serum do not induce a change in basal p62 expression when compared to healthy group; ICU low p62 (n = 9): group of patients from whom the serum induce a lower basal expression of p62; ICU high p62 (n = 55): group of patients from whom the serum induce an higher basal expression of p62.

An accumulation of p62 is a hallmark seen in neurodegenerative diseases and has also been described in critically ill patients. In order to reproduce the static measurement of p62 previously reported in the ICU patients^7,14^, the effect of patients’ serum on myotubes p62 expression was measured in a secondary analysis. On average, serum from the ICU patients significantly induced a higher expression of p62 in myotubes compared to serum of healthy volunteers (149.6 ± 46.6 vs. 122.6 ± 5.3 A.U., *p* = 0.0056; Mann-Whitney test exact *p* value; Fig. 4A). As for the autophagy flux measurements, a very large variation in serum induced p62 expression was observed in the patients, with a coefficient of variation for the patients of 31% compared to 4% for the controls. In accordance to the described rational to divide the cohort based on the level of autophagy flux, the patient cohort was divided into 3 groups based on the degree of the serum-induced increase in p62 expression e.g. ICU p62 non-responder (n = 29), ICU p62 high (n = 55) and ICU p62 low (n = 9). The threshold value used to split the cohort was defined from the measured basal p62 expression induced by serum from the healthy volunteers ± 2 SD (Fig. 4B). The autophagy flux was not different between the p62-based groups (*p* = 0.27). Interestingly, a trend was observed (*p* = 0.052, Kruskal-Wallis *p* value; Table 2) for a higher number of failing organs in the ICU p62 low group compared to the ICU p62 non-responder group (*p* = 0.057) and the ICU p62 high group (*p* = 0.07). No other clinical characteristics tended to be significantly different between the p62-based groups (Table 2). In the ICU p62 high group, the BCAA levels were 47%, 45% and 72% higher than in the healthy group, the ICU p62 non-responder group and the ICU p62 low group, respectively (Table 2). The same was observed for the EAA and NEAA, with levels significantly higher in the ICU p62 high group compared to the other groups (Table 2).

**Table 2:** Clinical parameters of patients group based on p62 basal expression.

	Healthy (n = 10)	ICU p62 non-responder (n = 29)	ICU p62 low (n = 9)	ICU p62 high (n = 55)	*p*
Age (years)	49 [44–53]	56 [48–65]	61 [45–78]	58 [53–62]	0.72
Sex (F/M)	(5/5)	(13/16)	(3/6)	(20/35)	0.76
BMI (kg/m^2^)	—	26.4 [24.3–28.5]	25.8 [23.9–27.6]	27.4 [25.6–29.2]	0.88
Length of Stay (days)	—	3.4 [2.4–4.5]	4.1 [1.5–6.8]	5.6 [3.8–7.3]	0.52
SOFA	—	6.2 [5.0–7.4]	8.7 [6.8–10.5]	6.3 [5.2–7.4]	0.11
APACHE		18.7 [15.6–21.7]	17.9 [15.4–20.3]	17.7 [15.6–19.9]	0.97
Number of organ failing	—	2.8 [2.4–3.1]	3.9 [3.2–4.6]	2.9 [2.5–3.3]	0.05
BCAA (µmol/L)	413^a,b^ [361–466]	422^a^ [344–499]	356^a^ [266–466]	611^b^ [540–682]	0.0001
EAA (µmol/L)	966^a^ [857–1075]	1001^a^ [816–1187]	865^a^ [624–1105]	1411^b^ [1251–1571]	<0.0001
NEAA (µmol/L)	2903^a,b^ [2708–3098]	2502^a,b,c^ [2251–2753]	2172^c^ [1805–2538]	3117^a^ [2881–3353]	<0.0001

Results are expressed as mean and 95% CI in bracket. BMI: Body mass index; SOFA: sequential organ failure assessment score; APACHE: Acute Physiology and Chronic Health Evaluation score; BCAA: branched chain amino acids (leucine, isoleucine and valine); EAA: essential amino acids (phenylalanine, threonine, tryptophan, methionine, lysine and histidine); NEAA: non-essential amino acids. Statistical difference were assessed by the non-parametric Kruskal-Wallis test with a ≠ b ≠ c, p < 0.05.

## Discussion

In the present study, serum of a subgroup of ICU patients (ICU blocker group) was able to block the flux of autophagy *in vitro* in human primary myotubes. This block was related to an accumulation of autophagosomes/autolysosomes, which indicates an impairment in the last steps of the autophagy process. The autophagy block was not associated to the type of nutritional support, either enteral or parenteral, nor related to amino acid or glucose levels. However, a lower aminoacidemia was observed in the ICU inducer group. In the second part, measurement of basal levels of p62, which mimics the static measurement of p62 previously reported *in vivo* in ICU patients, demonstrated a higher expression in the ICU patients compared to the healthy volunteers, but this was not related to any change in the autophagy flux. This higher accumulation of p62 was associated with higher levels of BCAA, EAA and NEAA and the group with lower expression tended to have more organ failings and a higher SOFA score.

Experimental animal models indicate that critical illness has an autophagy-deficient phenotype^9–11^ but importantly such models may not fully represent the complexity of critical illness induced organ failure in the clinical context^22–24^. The association between autophagy and organ failure is more unclear in humans, but available data such as the accumulation of autophagic substrates as p62 and LC3-II support the findings from experimental animal models^13^. To further characterize the effects on the autophagy process, to identify possible inter-individual differences in the autophagy flux as well as plausible systemic mediators, in the present study we utilize an *in vitro* model of human primary myotubes exposed to serum from well-characterized ICU patients. Serum from about 17% of the included ICU patients induced a block in the autophagy process in the myotubes. This observation suggests that circulating factors in serum from some ICU patients affected the autophagy process. p62 was significantly more colocalized with LC3 than with ubiquitin, which suggest an accumulation of autophagosomes/autolysosomes (LC3/p62 positive vacuoles) rather than an accumulation of protein aggregates (LC3/Ub positives vacuoles). Furthermore, the flux of autophagy was inversely correlated with the colocalization of p62 and LC3, which further indicates that the autophagy flux was blocked at the last step of the autophagy process, the fusion step. Unfortunately, since colocalization of LC3 to a lysosomal protein like LAMP1 was not performed, the present data is not able to differentiate between an accumulation of autophagosomes or autolysosomes.

The common nutrition strategy in the ICU clinic is an active and optimized nutrition treatment to minimize lean body and muscle loss. However, recent reports that early nutritional administration of energy and protein could interfere with the autophagy process make the picture more complicated since inhibition of the autophagy flux may participate in development of intensive care unit-acquired weakness and poor functional outcome^7,25^. Since posthoc analyses of these studies indicated protein nutrition to have the main affect, amino acids were analyzed in the serum in our study. Since no difference in serum amino acids levels were observed between the ICU non-responder group and the blocker group, the levels of amino acids could not explain the observed individual differences in blockage of the autophagy flux. Furthermore, earlier reports indicate that nutrition blocks autophagy at the initiation step^6,7^ and amino acids have been reported to primarily inhibited autophagy at the step of autophagosome formation^26^. For example, in an experimental model of rainbow trout (O. mykiss) dietary amino acids inhibited the autophagy flux through an activation of the mTOR pathway and a subsequent lowered the induction of autophagy with a concomitant decrease in autophagosome formation^27^. Hence, amino acids alone cannot explain the block in the later stage of autophagy observed in the ICU blocker group and most likely other circulating factors are involved in this block.

At the other end, starvation, especially amino acid starvation, is known to be a strong activator of autophagy, as confirmed in our autophagy flux assay when myotubes were incubated for 6 h in EBSS. In the ICU inducer group, a significant lower level of NEAA was observed and associated with a significant higher flux of autophagy to the level observed in the starvation control (EBSS). This is an important observation since it shows the potential of amino acid starvation to activate autophagy flux and suggests also an alternative explanation for the previous clinical studies, in that critical illness itself is inducing an autophagy block in some ICU patients and that semi-starvation in this group might overcome this block.

Serum of ICU patients induce on average, an accumulation of p62 (basal state) in human myogenic cells. This is a hallmark of autophagy inhibition and a higher expression of p62 was previously observed *in vivo* in skeletal muscle and liver of ICU patients^7,14^. The current p62 accumulation was however less dramatic than the levels in skeletal muscle and liver tissue from the ICU patients^7,14^. This might be explained by the fact that only the extrinsic effect of patient’s serum was tested and that myotubes were incubated for only 24 h. No difference in autophagy flux was observed between the groups based on p62 expression, which shows that expression of p62 in the absence of an autophagy inhibitor is not a good marker for autophagy dynamics. As recently shown in a “prolonged starvation model” where an increase in autophagy flux was associated with a restoration of p62 protein level via increased transcription^28^, this discrepancy between autophagy flux and p62 expression might be due to its regulation at the transcriptional level. The transcription level of p62 was not measured in our study. However, in the ICU high p62 group a higher basal expression of p62 was observed also in comparison to the p62 expression measured in human primary myotubes incubated with serum from the healthy volunteer and chloroquine. p62 is at the crossroad of the autophagy and the proteasome pathways and its expression level can either influenced or be influenced by both of these proteolytic pathways^29^. In mouse embryonic fibroblast cell lines, epoxomicin treatment (proteasome inhibition) induced a much higher increase in p62 expression than with chloroquine or bafilomycin A1 treatments (autophagy inhibition)^30^. However we did not assessed markers of proteotoxicity or the phosphorylation of p62 at the serine 409 induced by proteasome inhibition^31^ in our model but in future studies this needs to be further explored.

In summary, serum from ICU patients is able to trigger different responses in autophagy flux, reproducing the large variation in autophagy marker expression observed *in vivo* in ICU patients. A subgroup of critically ill ICU patients was identified whose serum was able to block autophagy in an *in vitro* model of primary human myotubes. Nutrition was most likely not a key factor since the individual levels of circulating amino acids could not explain the individual observed differences in the block. In addition, the block was observed in the later steps of the autophagy process with an accumulation of autophagosomes/autolysosomes which is unlike the normal step for nutrition to inhibit the autophagy flux. In patients with an increase in the autophagy flux, lower levels of amino acids were observed, suggesting a starvation like effect on the autophagy flux. All together the present study suggests that ICU patients have different phenotypes with respect to the autophagy flux and highlights the importance to identify the patients that might benefit or be harmed by early nutrition support. Only patients with a pre-existing critical illness-related autophagy block might benefit from withholding early nutritional support, whereas others are not and consequently underfed

## Methods

### Patients

Ninety-three consecutive critically ill patients treated in a mixed surgical-medical ICU (Karolinska University Hospital, Huddinge) were included between March 2011 and May 2011. This study was approved by The Regional Ethics Review Board in Stockholm (approval number 2011/133, amendment 2016/70) and a written informed consent was obtained from patients or close relatives to participate in the study. The exclusion criteria were pregnancy, an age below 18 years and the absence of informed consent. Six patients were also excluded due to insufficient volume of serum or the presence of HIV or hepatitis C. The inclusions process is presented in Additional File 1. Patient characteristics are shown in Table 1. A serum sample was obtained within the first 24 h after the patient’s admission to the ICU. Blood was allowed to coagulate for 60 minutes and serum was obtained by centrifuging at 2000 × G at room temperature. Serum from 10 individual healthy volunteers were purchased from 3H Biomedical, Uppsala, Sweden (Table 1). Samples were stored at −80 °C until analyses. The primary outcome of this study was the measurement of the autophagy flux. All experiments were performed in accordance with local and national regulations and guidelines.

### Cell culture

Human primary myoblasts were isolated freshly from vastus lateralis biopsies obtained from healthy volunteers. These cells were kindly provided by Thomas Gustafsson (Clinical Physiology, LABMED, Karolinska Institutet; Regional Ethics Review Board at Karolinska Institutet in Stockholm approval number 2012/173-31/3). All the participants were provided with information and a written informed consent was obtained from them. Myoblast were isolated by magnetic-activated cell sorting as previously described^32^. Once isolated, myoblasts were sub-cultured in T175 flask in growth media (Dulbecco’s Modified Eagle Medium (DMEM)/F12 GlutaMAX^TM^; Gibco®), supplemented with 20% fetal calf serum (FCS, heat-inactivated, certified; Gibco®) and 1% antibiotic-antimycotic (ABAM; Gibco®) at 37 °C and 5% CO_2_. For experiments, at 70% confluency myoblasts were detached with TrypLE^TM^ (Gibco®) and transferred to 96 well culture plates coated with collagen I bovine (Gibco®) at a concentration of 3.5 µg of collagen per cm^2^. The seeding density of the myoblasts for the experiments was 85 000 cells per cm^2^. At 90% of confluency, cell differentiation into myotubes was induced with differentiation medium (DMEM/F12 GlutaMAX supplemented with 2% FCS, 1% ABAM and 1% of Insulin-Transferrin-Selenium (ITS –G; Gibco®)). Medium was replaced every other day and myotubes differentiation was monitored by light microscopy. Myotubes used for the experiments were at day 14 of differentiation. Cells were washed once with serum free medium (DMEM/F12 GlutaMAX^TM^) and were then incubated in serum free media supplemented with 10% serum from the healthy volunteers or ICU patients. Serum samples from the different groups were placed randomly on the plates to achieve blinded analyses.

### Autophagy flux assay

Myotubes cultured in 96-well plates (Greiner Bio-One) were incubated for 24 hours at 37 °C and 5% CO_2_ with 10% serum from ICU patients (4 wells per patient). 6 h prior to the end of the 24 h incubation, half of the wells (2 per patient) were incubated with 50 µM of chloroquine (CQ), an autophagy inhibitor. Cells were fixed with 10% formalin and washed with PBS. Afterwards, cells were permeabilized with 0.1% Triton X-100 for 30 minutes at RT, blocked in blocking buffer (Odyssey® Blocking Buffer (PBS); LI-COR) for 2 hours, and incubated with a primary anti-p62 antibody (H00008878-M01, Abnova; 1:2500) overnight at 4 °C. After 3 * 5 min washing with PBS-TWEEN 0.1%, cells were incubated with a secondary antibody (IRDye® 800 CW, LI-COR; 1:1000) for 1 hours, and washed again with PBS-T. The plates were scanned on the Odyssey Scan, and were analyzed with Image Studio version 4.0. Once scanned, the plates were incubated with fluorescent dyes Draq5 (1:10000) and Sapphire700 (1:1000) to normalize the expression of p62 by cell number. The autophagy flux was calculated by the following calculation:

$$autophagy\,flux=(p62\,expression\,with\,CQ\,-p62\,expression\,without\,CQ)\ast 100$$

5 independent experiments were realized. Results were expressed as a percentage of the autophagy flux measured in untreated cells grown in regular DM (% control).

### Immunofluorescence

After the 24 h incubation with the serum, cells were directly fixed in 96 well glass bottom plates with 10% formalin for 15 min at RT. Cells were then permeabilized with pre-chilled methanol, 10 min at RT, and subsequently cells were incubated with 1% BSA for 1 hour. Afterwards cells were incubated with primary antibody overnight at 4 °C. The antibodies used in this study were anti-mouse P62 (H00008878-M01, Abnova; 1:200), anti-rabbit LC3-II (NB100-2220, Novus Biological; 1:500), anti-rabbit p62 (BML-PW9860, Enzo life sciences, 1:1000), anti-mouse ubiquitin (BML-PW8810, Enzo Life sciences; 1:1000). Cells were washed 3 × 5 min in PBS-Tween 0.1% and then incubated with secondary antibodies (Alexa 488-conjugated anti-mouse IgG and Alexa 568-conjugated anti-rabbit IgG) during 1 h at RT with a dilution of 1:1000 in BSA 1%. Finally, cells were washed 3 × 5 min in PBS-Tween 0.1% and then rinsed once in PBS and stored in PBS supplemented with 0.1% sodium azide. Plates were sealed and stored at +4 °C until imaging. Pictures were taken on an inverted fluorescent wide field microscope (Olympus IX-81) with an immersion oil objective (60X magnification, 1.35 numerical aperture (N.A)). To ensure a correct resolution in the z dimension, image stacks were taken with a z-step of 0.3 µm, according to the Nyquist criteria. Five 3 dimensional stacks were taken per condition with an image field size of 1376 × 1038 pixels on the x-y axis. The depth of the sample imaged was 9.6 µm.

### Image processing and analysis

The theoretical point spread function (PSF) of the 3D stack images was calculated from the Gibson and Lanne model in the ImageJ plugin PSF Generator (Biomedical Imaging Group (BIG), Switzerland). The deconvolution was performed using the Deconvolution Lab plugin for ImageJ (BIG, Switzerland) using the Richardson-Lucy algorithm. Background was removed using the rolling ball algorithm in ImageJ (10 pixel radius) and Gaussian filtered with a sigma (radius) of 2 pixels. The Pearson colocalization correlation coefficient (PCC), a pixel based colocalization algorithm, was calculated using the imageJ plugin coloc-2.

For the analysis of the particle size we used the image processed for the PCC calculation. These images were split in order to only keep the color channel of the protein of interest. These images were automatically threshold on ImageJ using the triangle algorithm. Then they were converted to mask and the module particle analysis of ImageJ was applied on the mask. From the resulting average particle size ($F$), the diameter ($d$) was calculated as follow: $d=2\surd (F/\pi )$. For all the immunostaining assays, samples were randomized and analyzed blindly and by automatic batch analysis. 2 independent experiments were realized.

### Serum amino acids concentration

Amino acid concentrations were analyzed by an HPLC method described previously^33^. Briefly, serum samples were deproteinized in 3% 5-sulfosalisylic acid-2-dihydrate (SSA) containing 200 μM norvaline as internal standard. Amino acids from the serum were analyzed using precolumn derivatization with orthophthaldialdehyde/3-mercaptopropionic acid on an HPLC system (Alliance, Waters 2690, fluorescence detector waters 474; Waters, Stockholm). The quantity of amino acid provided by the media of culture (DMEM/F12 GlutaMAX^TM^) was added to calculate the final concentration of amino acids per samples.

### Statistical analysis

All data were analyzed using GraphPad Prism 7.0. Normality of the data was assessed by the Shapiro-Wilk’s test. A one-way ANOVA was used for comparisons between the groups with normal distribution followed by a Bonferroni’s multiple comparison test to assess differences between groups. When the data values were not normally distributed, the statistical differences were assessed by a Kruskal & Wallis test followed by a Dunn’s multiple comparisons test. Correlation were assessed with the Spearman correlation test.

## Supplementary information

Figure S1

## Data Availability

The datasets used and/or analyzed during the current study are available from the corresponding author on reasonable request.
