# Supplementary material for: Autophagy flux in critical illness, a translational approach
Source: Sci Rep. 2019 Jul 24;9:10762. doi: 10.1038/s41598-019-45500-9 (PMC6656759; doi:10.1038/s41598-019-45500-9)
Supplement: Supplementary file 1 — Figure S1 [file 41598_2019_45500_MOESM1_ESM.pdf]

Autophagy flux in critical illness, a translational approach

Nicolas Tardif[1,2*], Franck Polia[2], Inga Tjäder[1,2], Thomas Gustafsson[3] and Olav Rooyackers[1,2]

[1]Division of Perioperative Medicine and Intensive Care, Karolinska University Hospital, Huddinge, Sweden

[2]Anesthesiology and Intensive Care, Department of Clinical Science Intervention and Technology (CLINTEC), Karolinska Institutet, Huddinge, Sweden

[3]Division of Clinical Physiology, Department of Laboratory Medicine, Karolinska Institutet, and Unit of Clinical Physiology, Karolinska University Hospital, Stockholm, Sweden

[*]Corresponding author: Nicolas Tardif, PhD

Anesthesiology and Intensive Care, CLINTEC, Karolinska Institutet 141 86 Stockholm

Phone: +46 8 585 838 77; email: nicolas.tardif@ki.se

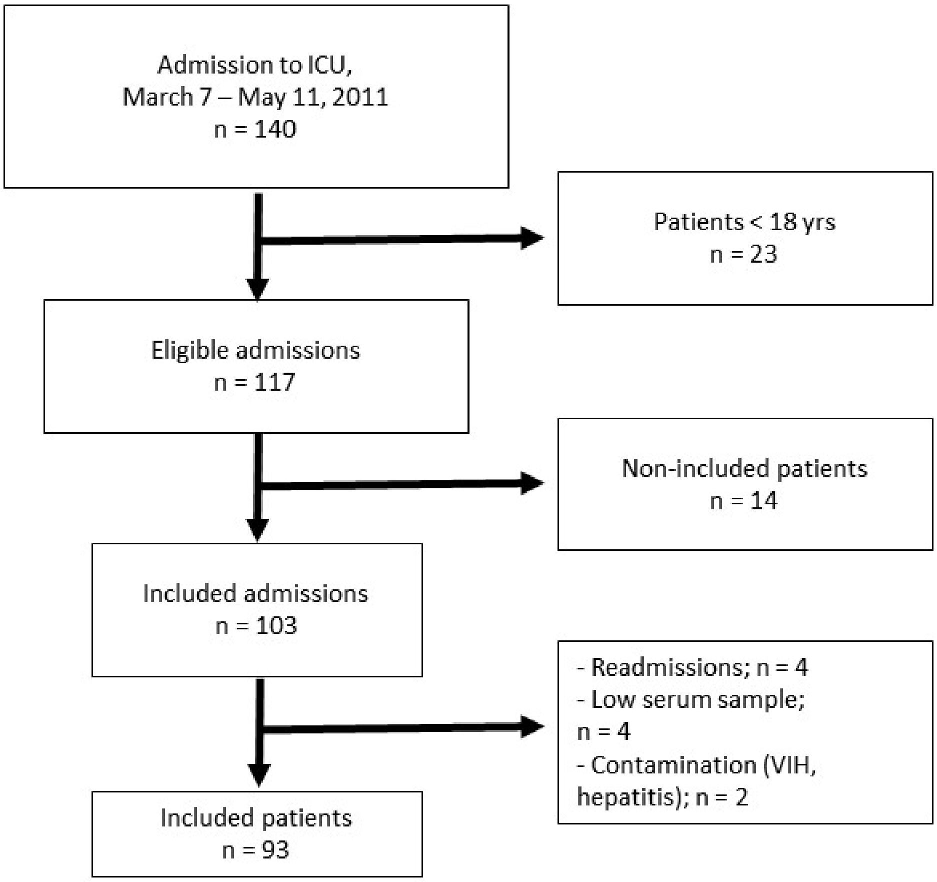

Figure S1. Flowchart of the inclusion selection
